# Conversion between duplicated genes generated by polyploidization contributes to the divergence of poplar and willow

**DOI:** 10.1186/s12870-022-03684-9

**Published:** 2022-06-17

**Authors:** Jianyu Wang, Lan Zhang, Jiaqi Wang, Yanan Hao, Qimeng Xiao, Jia Teng, Shaoqi Shen, Yan Zhang, Yishan Feng, Shoutong Bao, Yu Li, Zimo Yan, Chendan Wei, Li Wang, Jinpeng Wang

**Affiliations:** 1 School of Life Sciences, and Center for Genomics and Computational Biology, North China University of Science and Technology, Tangshan, 063000 Hebei China; 2 University of Chinese Academy of Sciences, Beijing, 100049 China; 3 State Key Laboratory of Systematic and Evolutionary Botany, Institute of Botany, Chinese Academy of Science, Beijing, 100093 China

**Keywords:** Poplar, Willow, Polyploidization, Duplicated genes, Gene conversion

## Abstract

**Background:**

Gene conversion has an important effect on duplicate genes produced by polyploidization. Poplar (*Populus trichocarpa*) and willow (*Salix brachista*) are leading models and excellent green plants in the Salicaceae. Although much attention has been paid to the evolution of duplicated genes in poplar and willow, the role of conversion between duplicates generated from polyploidization remains poorly understood.

**Results:**

Here, through genomic synteny analyses, we identified duplicate genes generated by the Salicaceae common tetraploidization (SCT) in the poplar and willow genomes. We estimated that at least 0.58% and 0.25% of poplar and willow duplicates were affected by whole-gene conversion after the poplar-willow divergence, with more (5.73% and 2.66%) affected by partial-gene conversion. Moreover, we found that the converted duplicated genes were unevenly distributed on each chromosome in the two genomes, and the well-preserved homoeologous chromosome regions may facilitate the conversion of duplicates. Notably, we found that conversion maintained the similarity of duplicates, likely contributing to the conservation of certain sequences, but is essentially accelerated the rate of evolution and increased species divergence. In addition, we found that converted duplicates tended to have more similar expression patterns than nonconverted duplicates. We found that genes associated with multigene families were preferentially converted. We also found that the genes encoding conserved structural domains associated with specific traits exhibited a high frequency of conversion.

**Conclusions:**

Extensive conversion between duplicate genes generated from the SCT contributes to the diversification of the family Salicaceae and has had long-lasting effects on those genes with important biological functions.

**Supplementary Information:**

The online version contains supplementary material available at 10.1186/s12870-022-03684-9.

## Background

As one of the two mechanisms of homologous recombination, gene conversion involves the unidirectional transfer of one gene (or DNA segment) to its paralogous counterpart [1–3]. Gene conversion can occur not only between alleles on homologous chromosomes, but also between paralogs on homoeologous chromosomes produced by polyploidization, and between paralogs created by other types of genomic duplication, e.g., transposon activity or tandem duplications. Research has revealed that gene conversion has affected the evolution of many duplicated genes produced by polyploidizations [1, 4–6]. Gene conversion between duplicated genes (or homoeologous chromosomes) generated from WGD events has been identified in the genomes of *Poaceae* [1, 7, 8], *Arachis hypogaea* [9], *Gossypium* [5], *Brassica campestris*, and *Brassica oleracea* [10]. In addition, gene conversion between duplicated genes is frequent and long-lasting, as demonstrated by rice homoeologous chromosomes 11 and 12, which were produced from common tetraploidization events in grasses [1, 8, 11, 12].

Poplar (*Populus trichocarpa*, 2*n* = 2*x* = 38) and willow (*Salix brachista*, 2*n* = 2*x* = 38) are excellent green plants in the Salicaceae. They have a wide ecogeographic range, spanning the entire northern hemisphere [13, 14], and the global planting area exceeds 80 million hectares [15]. Poplar and willow both have great economic and ecological value [16] and are commonly used for shelterbelts, timber, and landscape forests, as well as even erosion control and wastewater treatment [17–20]. Poplar is often considered the leading tree of family Salicaceae, mainly due to its small genome size, rapid growth, and easy clonal reproduction [21–24]. Willow has similar genomic characteristics to poplar [25], making it an ideal model system for investigating intraspecific divergence and adaptive evolution in alpine species [26]. Mainly due to their biological and economic importance, the genomes of *P. trichocarpa* and *S. brachista* have been sequenced [26, 27]. Currently, although much attention has been given to the evolution of duplicated genes in poplar and willow, a comprehensive analysis of the conversion between duplicated genes is still lacking.

**Fig. 1:**
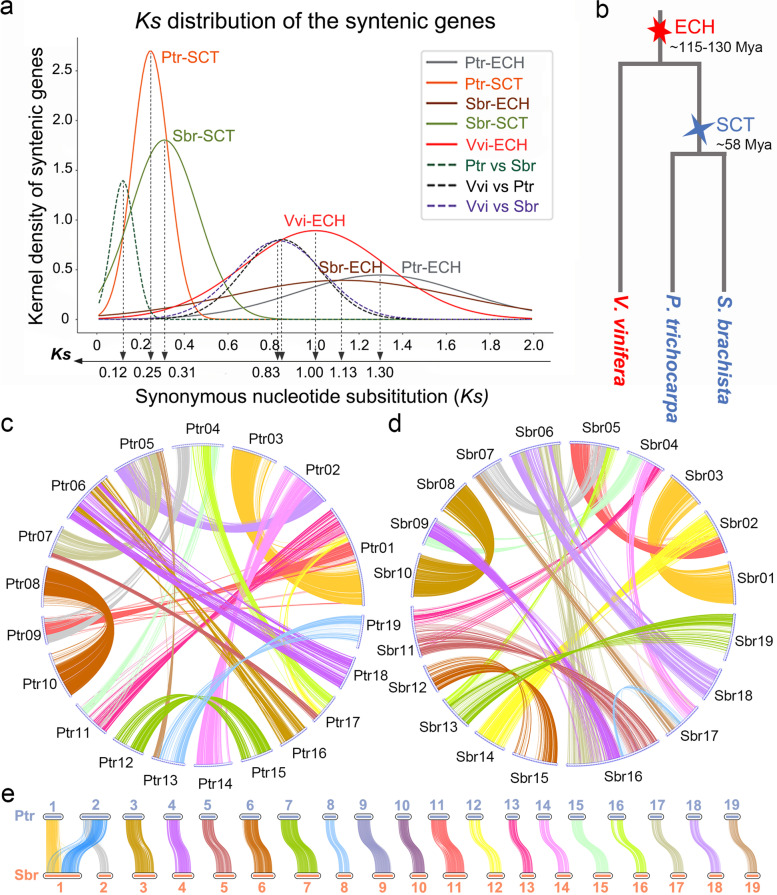
Inference of paralogous and orthologous genes. ECH or *gamma*, core-eudicot-common hexaploidization; SCT, Salicaceae common tetraploidization. **a** *Ks* analyses of colinear genes within and between genomes. Continuous curves show the *Ks* distribution within a genome, and broken curves show the *Ks* distribution between genomes. **b** Phylogenic tree of *V. vinifera*, *P. trichocarpa* and *S. brachista.* **c** Paralogs in *P. trichocarpa*; the connecting line represents a pair of duplicated genes. **d** Paralogs in *S. brachista*. **e** Orthologs between *P. trichocarpa* and *S. brachista*. (PDF 2,196 kb)

Polyploidization refers to the duplication of all chromosomes within a cell and is also called whole-genome duplication (WGD); this process is widespread in the evolutionary history of green plants (Viridiplantae) [28–31]. Polyploidization can provide basic genomic material for species evolution and can trigger speciation and diversification in the angiosperms [32–37]. Following polyploidization, an enormous number of duplicated genes are generated, leading to genome instability [1], which is characterized by extensive chromosomal rearrangements and a large number of duplicate gene losses [9, 38–42]. The retained duplicated genes generated from WGD events may give rise to novel functions, the subdivision of ancestral functions, or a mixture of both through nucleotide mutations [43–46]. In contrast to nucleotide mutation, DNA recombination enables duplicate genes to interact with each other and ultimately result in genetic innovation [8, 47, 48]. The common ancestral genomes of poplar and willow undergone recursive WGD events. First, by definition, all gymnosperms, including poplars and willows, have necessarily experienced the core-eudicot-common hexaploidization event (ECH or *gamma*) that occurred at about 115–130 MYA (Fig. 1b) [49–51]. Poplar and willow then recently underwent a common tetraploidization event, which we refer to as Salicaceae common tetraploidization (SCT), which occurred ~ 58 million years ago (MYA) [19].

After the shared SCT event, thousands of duplicated gene pairs were generated in the poplar and willow genomes, providing innovative material for the evolution and diversification of the Salicaceae, as revealed in previous studies [19, 27, 52, 53]. These duplicates were symmetrically distributed on two sets of subgenomes related to the SCT event, suggesting autopolyploidy [54, 55]. Autopolyploids are formed by duplication of the genomes with relatively balanced subgenomic duplicates, whereas allopolyploids arise from crosses between different species with some independent evolutionary history, the distribution of subgenomic duplicates is usually unbalanced [55]. Ancient autopolyploidy shows a slower rate of genomic evolution, as demonstrated in previous studies on the genomes of soybean and kiwifruit [42, 56]. The poplar genome also exhibits a slower evolution rate compared to that of willow, and both rates are substantially lower than those of *Arabidopsis thaliana* and *Oryza sativa* [19, 25, 27, 55]. It would be interesting to know whether the slower evolutionary rate of the poplar genome is related to the gene conversion between duplicated genes.

Here, by comparing the latest high-quality genomes of poplar and willow, we aim to identify the paralogous and orthologous genes generated from the SCT and the divergence of these two species, respectively, to assess the scale and pattern of conversion between duplicated genes. We also aim to explore the factors that influence the occurrence of conversion and its impact on genome evolution, expression, and biological function.

## Results

### Inference of paralogous and orthologous genes

Through intragenomic comparison analyses (Additional file 1: Fig. S1-2) and homology searches with BLASTP [57] and ColinearScan [58], we identified the paralogous and orthologous genes within and between the *P. trichocarpa* and *S. brachista* genomes (Additional file 2: Table S1). In *P. trichocarpa,* we identified 1,381 blocks with ≥ 4 colinear genes, containing 24,073 colinear gene pairs. However, using the same criteria, we only identified 1,305 blocks containing 19,512 colinear gene pairs in the *S. brachista* genome. This indicates that the *P. trichocarpa* genome has a highly conserved intragenomic homology compared to that of *S. brachista* and that *S. brachista* underwent more chromosomal rearrangements after divergence from *P. trichocarpa*. Furthermore, the synonymous nucleotide substitution rates (*Ks*) within blocks were analyzed to distinguish blocks related to different polyploidization events [42]. We identified the blocks related to the SCT in the *P. trichocarpa* and *S. brachista* genomes. We observed a clear bimodal structure in the *Ks* distribution of colinear genes within the genome (Fig. 1a, Additional file 1: Fig. S3-4, Additional file 2: Table S2). There was a small *Ks* peak at ~ 0.25 related to the SCT and a larger *Ks* peak at ~ 1.30 related to the ECH in *P. trichocarpa*. In *S. brachista*, the small *Ks* peak at ~ 0.31 was related to the SCT, and the larger *Ks* peak at ~ 1.13 was related to the ECH. Finally, we inferred the duplicate genes generated from the SCT in *P. trichocarpa* and *S. brachista* (Fig. 1c-d). We identified 12,203 duplicated genes (35.4% of the whole genome) located in 138 blocks generated from the SCT in *P. trichocarpa* and 9,280 duplicated genes (30.9% of the whole genome) located in 162 blocks generated by the SCT in *S. brachista*.

To identify the orthologs between genomes, we performed intergenomic comparisons between *P. trichocarpa* and *S. brachista* (Additional file 2: Table S1). We found at least 2,423 blocks involving 46,684 colinear gene pairs between the *P. trichocarpa* and *S. brachista* genomes. In addition, we found that *P. trichocarpa* and *S. brachista* have well preserved regions of collinear genes due to their short divergence time, and the median *Ks* of homologous genes on these collinear were all floating around 0.12 (Fig. 1a, Additional file 1: Fig. S5). Based on the median *Ks* of ~ 0.12 for anchored gene pairs located in blocks, we then identified 18,861 orthologs located in 105 blocks between the *P. trichocarpa* and *S. brachista* genomes (Fig. 1e).

### Construction of homologous gene quartets

**Fig. 2:**
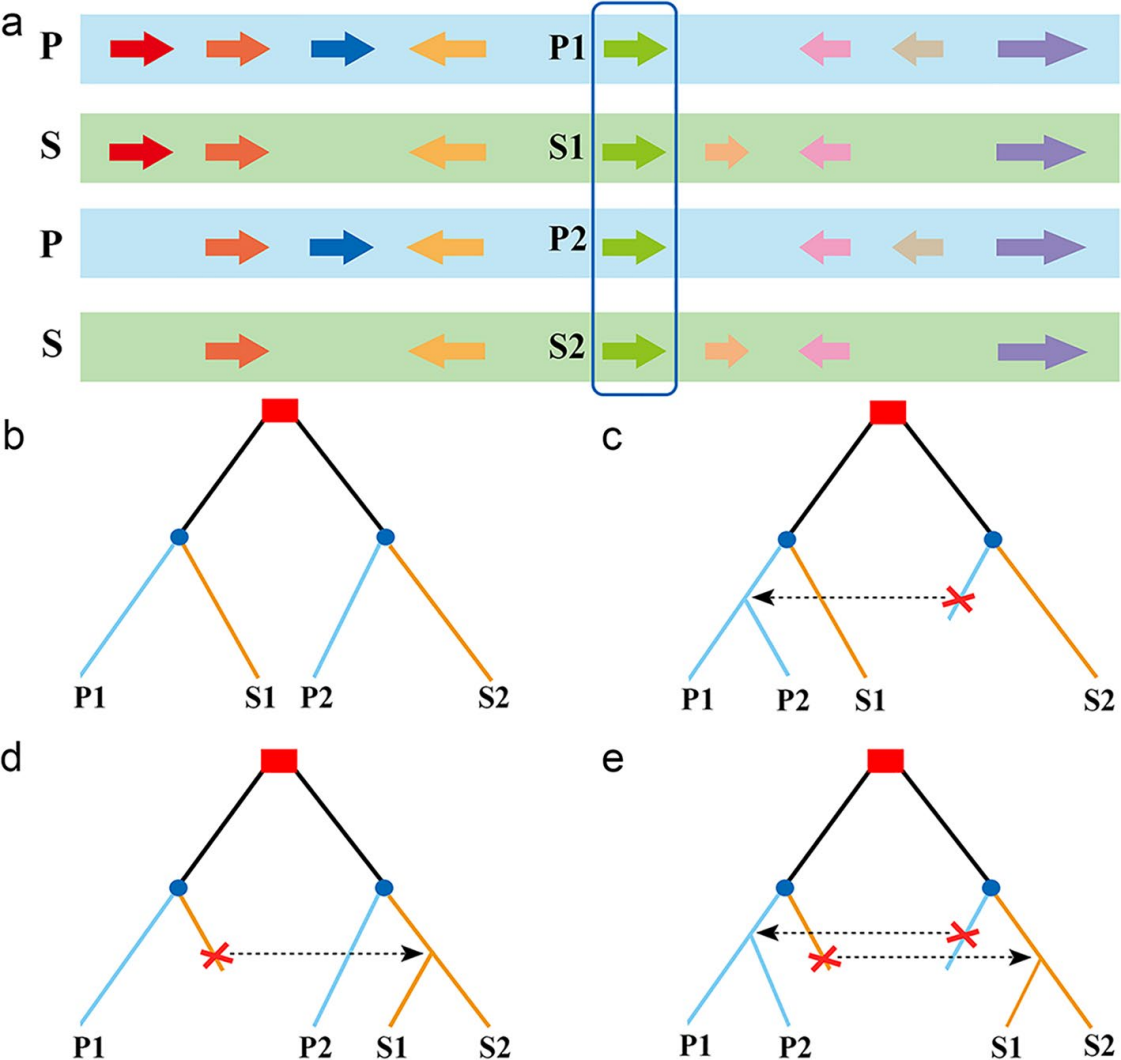
Definition of homologous gene quartets and inference of conversion between duplicates. **a** Definition of homologous gene quartets. Homoeologous chromosomal regions of *P. trichocarpa* and *S. brachista* (P and S) are represented by rectangles with different colors. Arrows indicate genes, and the same color indicates homologous genes. A homologous gene quartet consists of paralogous P1 and P2 and their respective orthologous genes S1 and S2. **b-e** Inference of conversion. The red squares indicate the SCT, and the blue circles indicate the divergence of *P. trichocarpa* and *S. brachista*. Expected phylogenetic relationships if no paralogs are affected by conversion are shown in **b**, P2 converted by P1 is shown in **c**, S1 converted by S2 is shown in **d**, and both paralogs affected by conversion are shown in **e**. (PDF 606 kb)

To deduce possible gene conversion between duplicate gene pairs generated by SCT, we used the above identified paralogous and orthologous gene pairs to define homologous gene quartets between *P. trichocarpa* and *S. brachista*. In *P. trichocarpa*, if one pair of duplicated chromosomal segments was established by the WGD of the common ancestor, we could always find a pair of duplicated genes P1 and P2 and their respective orthologous genes S1 and S2 in *S. brachista*. These four homologous genes were defined as a homologous gene quartet (Fig. 2a). Expectedly, the similarity of orthologous gene pairs is higher than that of the paralogous gene pairs in each quartet because orthologs are separated later than paralogs. However, if the paralogous genes are affected by gene conversion, their similarity will be changed, and the topology of the gene trees of the quartets will be different from what is expected (Fig. 2b-e) [11]. We constructed 4,813 quartets between the *P. trichocarpa* and *S. brachista* genomes, according to the definition of a homologous gene quartet (Additional file 2: Table S3-5).

### Conversion between SCT-related duplicated genes in poplar and willow

**Fig. 3:**
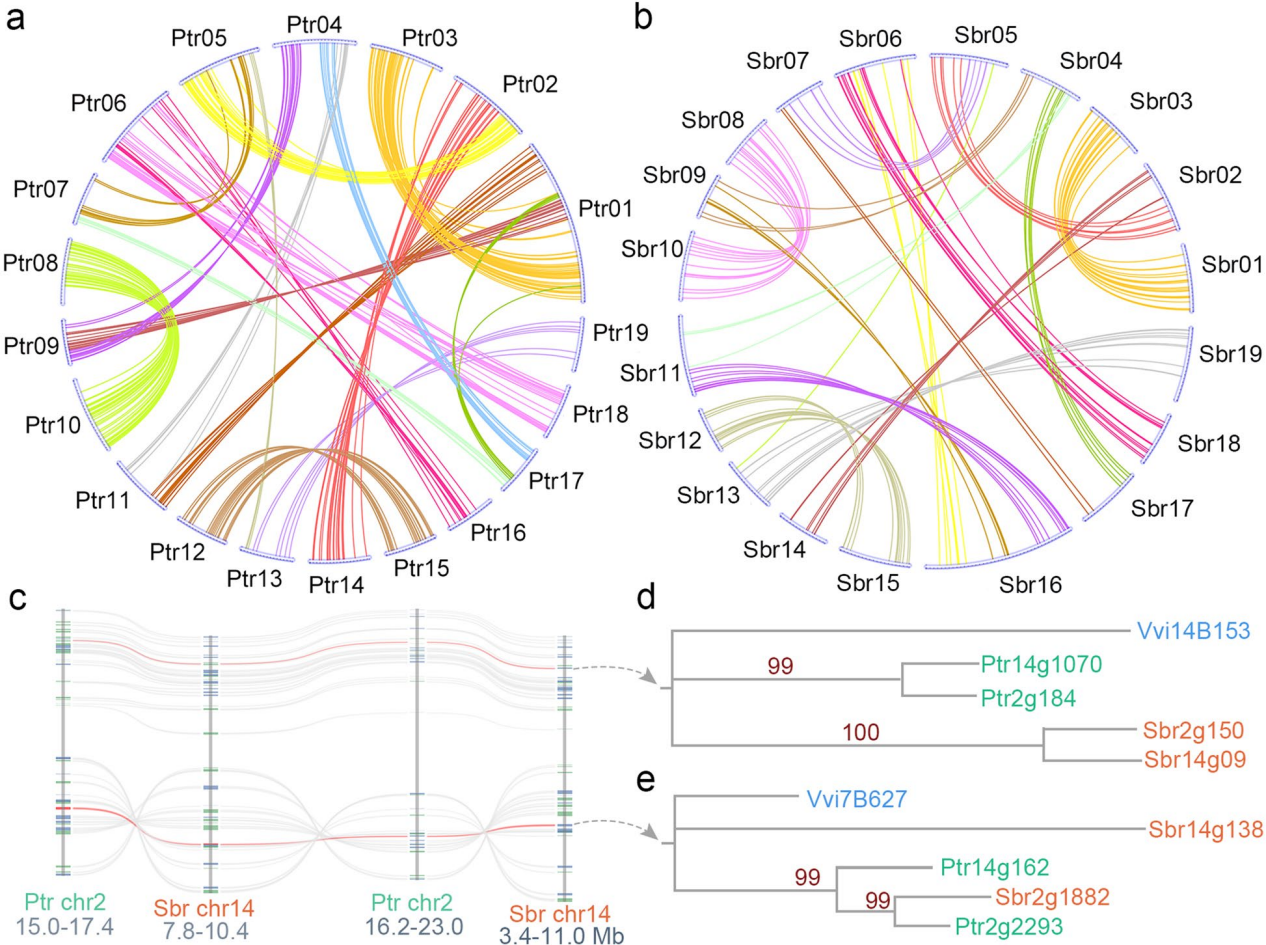
Converted duplicates and an example of conversion. **a** Converted paralogs in *P. trichocarpa*. Each converted duplicated gene is connected by the colored curves. **b** Converted paralogs in *S. brachista*. **c** Quartets in local colinear regions from *P. trichocarpa* and *S. brachista*. Duplicates affected by conversion connected by red lines. **d** Two pairs of duplicated genes in *P. trichocarpa* and *S. brachista* affected by WCV. **e** Topological tree affected by partial-gene conversion. The nucleotide site 454 to 494 of *Ptr14g1628* were partially converted by its paralog *Ptr02g2293*. (PDF 1,683 kb)

We aligned homologous gene sequences in each quartet with ClustalW software [59] to detect the duplicated genes that may be affected by gene conversion. After eliminating the highly divergent quartets with gaps accounting for > 50% of the alignment length or those with an amino acid identity between any compared gene pairs less than 40%, we ultimately obtained 4,813 reliable quartets for further inferring gene conversion. For quartets, we employed two methods to infer conversion. One is based on synonymous nucleotide substitutions (*Ks*) as a similarity measure to detect the paralogs with whole-gene conversion (WCV), and the other uses a combination of dynamic programming and phylogenetic analysis to detect paralogs with partial-gene conversion (PCV) [8, 48]. In *P. trichocarpa*, we found that 6.32% (304/4,813) of the paralogs were converted after the divergence of *P. trichocarpa* and *S. brachista*, in which 0.58% (28/4,813) of the paralogs were affected by WCV and 5.73% (276/4,813) were affected by PCV (Fig. 3a). In *S. brachista*, we found that 2.91% (140/4,813) of the paralogs were converted after divergence from *P. trichocarpa*, of which 0.25% (12/4,813) were affected by whole-gene conversion and 2.66% (128/4,813) were affected by partial-gene conversion (Fig. 3b, Additional file 2: Table S3). By comparing the converted paralogs in the two genomes, we found that the conversion rate of *P. trichocarpa* was 6.31%, which was twice that of *S. brachista* at 2.91%.

To better understand the patterns of gene conversion, we present two conversion examples that are located on homologous chromosome regions produced by the SCT in *P. trichocarpa* and *S. brachista* (Fig. 3c). We inferred that the paralogs from the quartet containing *Ptr14g1070*, *Ptr02g1847*, *Sbr14g0921*, and *Sbr02g1500* were affected by WCV in both *P. trichocarpa* and *S. brachista*, and the gene conversion events can be reflected by the changes in the topological tree compared to their expected structure (Fig. 3d). For the quartet containing *Ptr14g1628*, *Ptr02g2293*, *Sbr14g1384*, and *Sbr02g1882*, we inferred that *Ptr14g1628* was partially converted by its paralogous gene *Ptr02g2293* from bases 454 to 494, resulting in *Ptr14g1628* being more similar to *Ptr02g2293* than to its orthologous gene *Sbr14g1384* (Fig. 3e).

### Well-preserved chromosomal regions facilitate the conversion

Previous studies reported that paralogous genes near chromosomal termini are more frequently affected by gene conversion [1, 8, 47]. To check whether this rule exists in Salicaceae, we first divided poplar and willow chromosomes into 10 intervals based on length, and calculated the rates of converted duplicates within each interval (Additional file 2: Table S6). We found that a total of 53 duplicates (4.31% of the total duplicates, 53/1,231) were converted in the first 10% of *P. trichocarpa* chromosomes and 73 duplicates (4.81% of the total duplicates, 73/1,517) were converted in the last 10%, and 469 duplicates (4.60% of the total duplicates, 469/10,195) were converted in the other regions; a total of 21 duplicates (2.66% of the total duplicates, 21/790) were converted in the first 10% of *S. brachista* chromosomes and 47 duplicates (4.10% of the total duplicates, 47/1,145) were converted in the last 10%, and 199 duplicates (2.52% of the total duplicates, 199/7,872) were converted in the other regions. We compared the rate of converted genes at the ends (anterior and posterior) of *P. trichocarpa* and *S. brachista* chromosomes with other regions and found no significant differences in converted rates in these regions (*P* value = 0.974 and 0.132, one-way analysis of variance). This statistic shows no bias toward the conversion of duplicates near the end of chromosomes in *P. trichocarpa* and *S. brachista*.

**Fig. 4:**
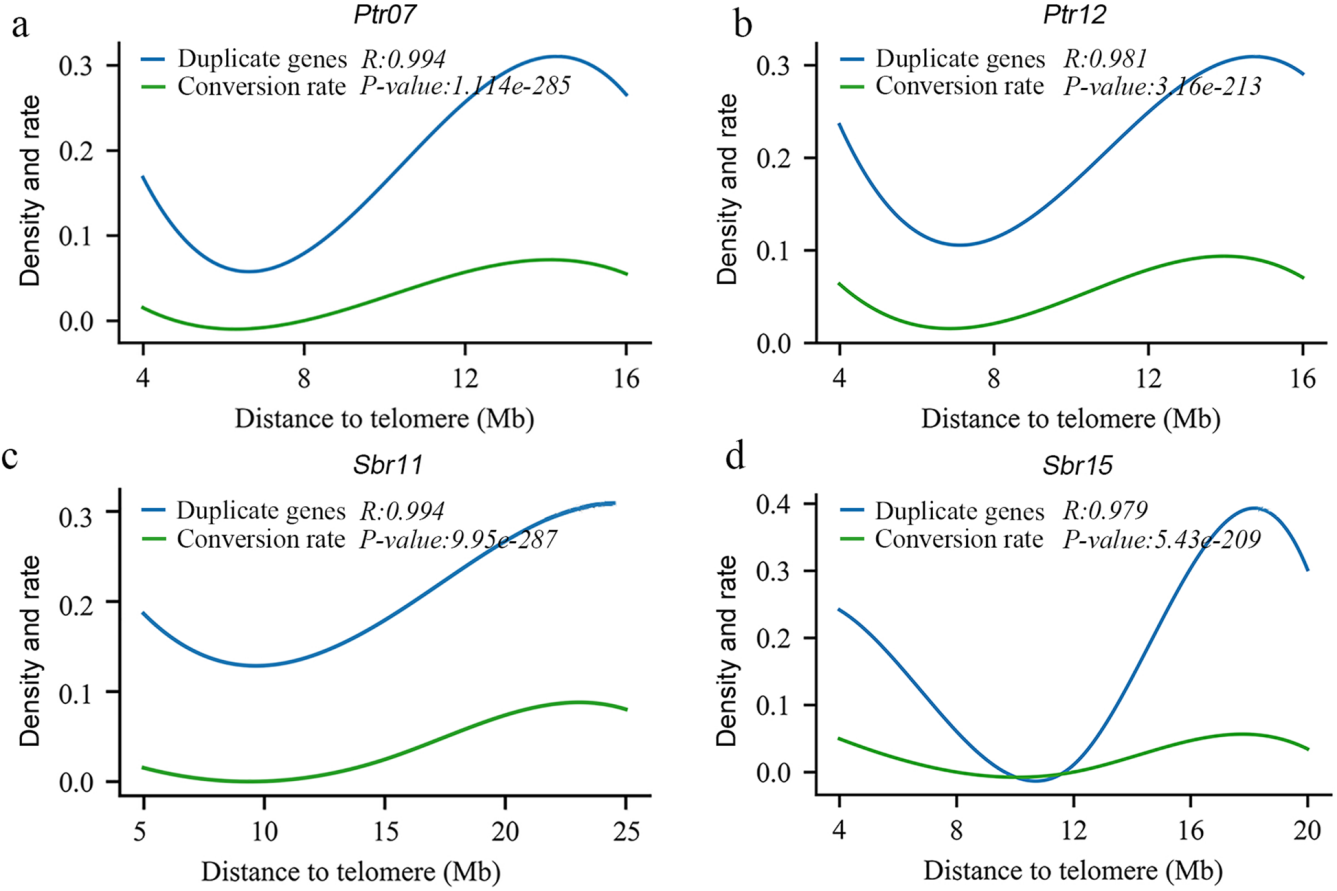
The correlation between conversion and the density of duplicated genes on selected chromosomes. **a-d** The correlation patterns of chromosomes 7 and 12 in *P. trichocarpa* and chromosomes 11 and 15 in *S. brachista*. The Y-axis indicates the density of duplicated genes (blue lines) and conversion rate (green lines) for selected chromosomes in *P. trichocarpa* and *S. brachista*. The X-axis indicates the distance of the duplicated or converted genes from the chromosome termini. (PDF 503 kb)

Furthermore, in the above results we observed that the distribution of duplicated genes and converted duplicates was different between *P. trichocarpa* and *S. brachista* chromosomes. Meanwhile gene conversion usually occurs between duplicated genes generated by polyploidization. Therefore, to investigate whether the uneven distribution of gene conversion across the chromosomes was due to the uneven distribution of duplicated genes, we characterized the correlation between duplicate gene density and the conversion rate for each chromosome (Fig. 4a-d, Additional file 1: Fig. S6-7, Additional file 2: Table S7-8). In *P. trichocarpa*, we found that 68% (13 of 19) of the chromosomes had a positive correlation between the density of duplicates and the conversion rate, of which 8 chromosomes were significantly correlated. Chromosomes 7 and 12 in *P. trichocarpa* showed a frequency of gene conversion that was significantly positively correlated with the density of duplicate genes. Similar patterns were also found in *S. brachista*; we found that 47% (9 of 19) of the chromosomes had a correlation between the density of duplicates and the conversion rate, of which 4 chromosomes were significantly correlated. Chromosomes 11 and 15 in *S. brachista* showed a frequency of gene conversion that was significantly positively correlated with the density of duplicate genes. This statistical analysis indicates that the well-preserved chromosomal regions often retained more duplicated genes and showed a high frequency of conversion, which facilitates the conversion of duplicate genes.

### Conversion contributes to the divergence of P. trichocarpa and S. brachista

Gene conversion homogenizes the sequences of paralogous gene pairs, making the paralogs seem younger than orthologs when examining the divergence of species. Therefore, the *Pn* and *Ps* (*Pn* and *Ps* refer to the *Ka* and *Ks* values corrected by Jukes-Cantor model, see Methods for details) between converted paralogs would be smaller than those between nonconverted paralogs (Table 1). To investigate the relationship between conversion and the genome evolutionary rate, we first compared the *Ps* values of converted and nonconverted duplicated genes in the two genomes. We found an average *Ps* of 0.220 for converted paralogs, which was smaller than the average *Ps* of 0.264 found for nonconverted paralogs in *P. trichocarpa* (*P* value = 0.394), while in *S. brachista*, an average *Ps* of 0.314 was found for converted paralogs, which was smaller than the average *Ps* of 0.347 found for nonconverted paralogs (*P* value = 0.103). This comparison only indicates that the duplicate gene pairs become more similar due to conversion but cannot be regarded as evidence of a slowing down of the rate of evolution. The main reason is that the evolutionary distance of paralogous gene pairs has been altered by conversion, as demonstrated in previous studies [1, 8]. Therefore, we further compared the *Ps* values of converted and nonconverted duplicates between genomes. We found an average *Ps* of 0.461 for converted orthologs, which was significantly larger than the average *Ps* of 0.163 for nonconverted orthologs (*P* value = 1.10E-55). Through these comparisons, we conclude that the conversion makes duplicate pairs of genes more similar to each other, likely contributing to the conservation of these sequences, but it essentially accelerates the evolutionary rate and the divergence of *P. trichocarpa* and *S. brachista*.

**Table 1:** Nucleotide substitution rates of quartets in the *P. trichocarpa* and *S. brachista* genomes

	Converted genes		Nonconverted genes	*P* value (T test)
Paralogs in *P. trichocarpa*	*Pn*	0.075	0.085	0.587
	*Ps*	0.220	0.264	0.394
	*Pn*/*Ps*	0.380	0.343	0.432
Paralogs in *S. brachista*	*Pn*	0.113	0.130	0.577
	*Ps*	0.314	0.347	0.103
	*Pn*/*Ps*	0.336	0.295	0.197
Orthologs between *P. trichocarpa* and *S. brachista*	*Pn*	0.231	0.058	2.02E-46
	*Ps*	0.461	0.163	1.10E-55
	*Pn*/*Ps*	0.523	0.328	3.61E-11

Furthermore, we used *Pn*/*Ps* ratios to examine whether the selection pressure of duplicated genes was changed under conversion (Table 1). First, we found that the average *Pn*/*Ps* ratio of converted paralogous genes in *P. trichocarpa* was 0.380, which is slightly larger than that of nonconverted paralogous genes at 0.343 (*P* value = 0.432). Similar to *P. trichocarpa*, we also found that the average *Pn*/*Ps* ratio of converted paralogous genes in *S. brachista* was 0.336, which was also slightly larger than that of nonconverted paralogous genes at 0.295 (*P* value = 0.197). This result seems to indicate that conversion does not change the selection pressure on duplicate genes. However, we note that only relying on the *Pn*/*Ps* ratios of paralogous gene pairs within genomes cannot detect the true change in selection pressure because conversion has changed the nonsynonymous and synonymous nucleotide substitution rates, which could also lead to their ratio being distorted. Therefore, we further used the *Pn*/*Ps* ratios of orthologs to determine the actual differences in selection pressure. We found that the average *Pn*/*Ps* ratio of converted orthologous genes between the two genomes was 0.523, which was significantly larger than the average *Pn*/*Ps* ratio of nonconverted orthologous genes at 0.328 (*P* value = 3.61E-11). These comparisons suggest that conversion may reduce the purifying selection pressure on genes and play a role in relieving the evolutionary pressure on duplicated genes.

### Conversion and gene expression patterns

To discover the potential relationship between conversion and gene expression, we compared the changes in expression patterns between converted and nonconverted duplicates by analyzing the transcriptomes of poplar and willow (Additional file 2: Table S9). For the *P. trichocarpa* secondary xylem, we found that 52.16% (133/255) of the converted duplicate gene pairs had differential expression greater than a twofold change, which was slightly less than the rate of nonconverted duplicates at 57.08% (2,906/5,091). Similarly, we found that 52.63% (70/133) of the converted duplicates had differential expression greater than a twofold change, which was also slightly less than the rate of nonconverted duplicates at 57.19% (2,632/4,602) in *S. brachista* leaves (Additional file 2: Table S10). This finding suggests that the converted genes had smaller gene expression differences than the nonconverted genes in *P. trichocarpa* and *S. brachista*. Furthermore, by comparing the TPM (Transcripts per million, Transcripts Per Kilobase of exon model per Million mapped reads) differences between converted and nonconverted duplicated gene pairs, we found that the mean TPM of converted genes was 35.69, which was significantly smaller than that of nonconverted genes at 47.04 (*P* value = 0.03, T test) in *P. trichocarpa* secondary xylem. Similar changes in gene expression patterns were also detected in *S. brachista* leaves (Additional file 2: Table S11). Therefore, conversion may result in duplicated gene pairs showing more similar expression patterns than nonconverted pairs.

### GO analysis of the duplicated genes

**Fig. 5:**
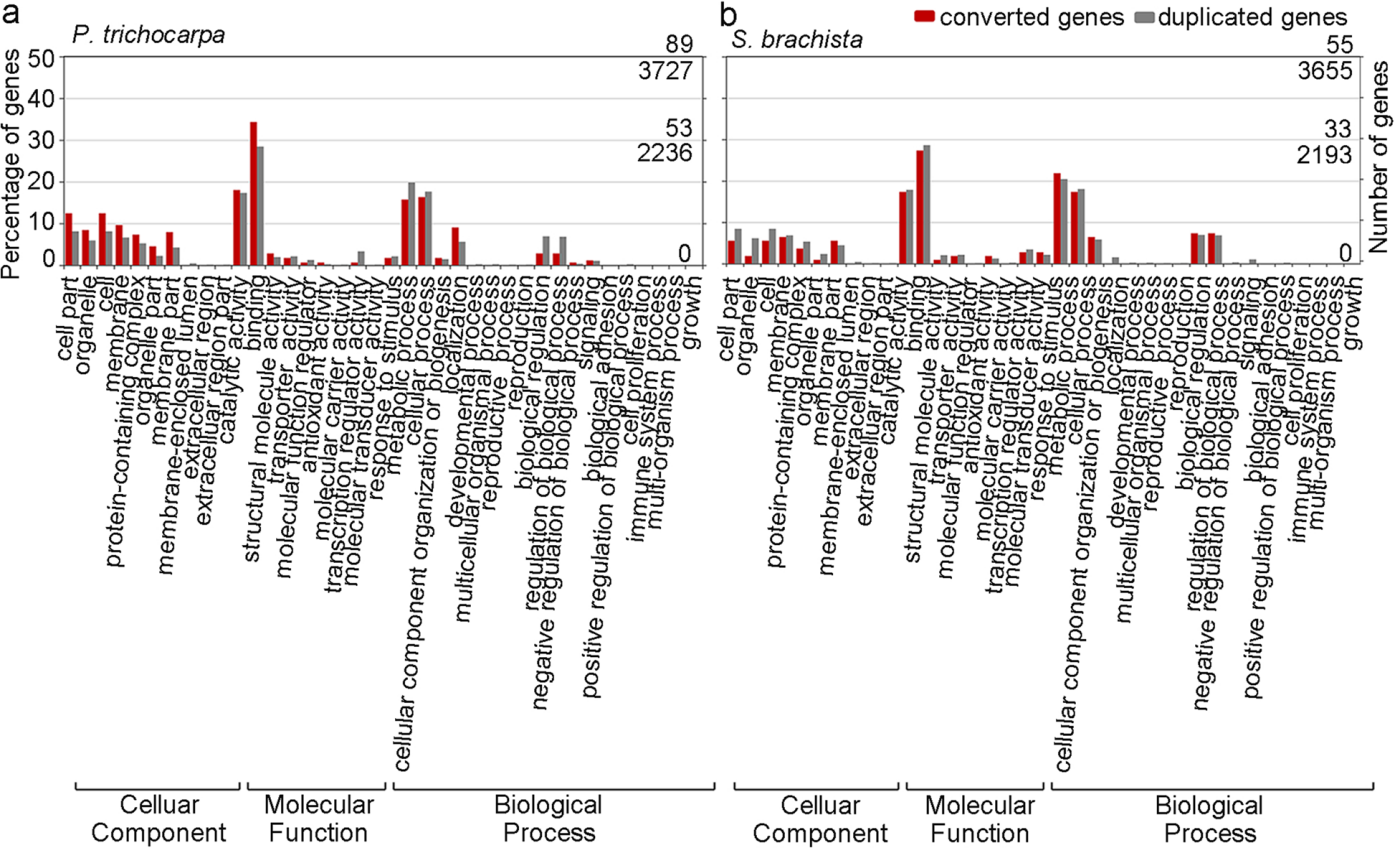
Histograms of Gene Ontology (GO) statistics for converted genes and nonconverted genes. **a** GO analyses of the duplicated genes in the *P. trichocarpa* genome. The X-axis shows the GO terms; the Y-axis shows the percentages generated from the number of converted genes in each GO term divided by the total number of converted genes (colored red) and the number of duplicated genes in each GO term divided by the total number of duplicated genes (colored gray). **b** GO analyses of the duplicated genes in the *S. brachista* genome. (PDF 568 kb)

The biological function of a gene may correlate with its chance of being converted. To investigate the correlation between conversion and gene function, we identified the associated terms for duplicated genes in the poplar and willow genomes by performing Gene Ontology (GO) analysis (Additional file 2: Table S12). In *P. trichocarpa*, we found that genes associated with cellular components usually accounted for a large proportion of all duplicated genes, and these genes are often affected by conversion. For the functions of cell and cell part, we found additional secondary-level terms in 12.4% of the converted genes, which is significantly higher than their expected percentage of 8.1% in duplicated genes (*P* value = 0.041) (Fig. 5a, Additional file 2: Table S13). Similarly, we found that the genes involved in binding were more frequently affected by conversion and made up a large proportion of duplicated genes (Fig. 5a). In contrast, the genes related to biological process functions, which are encoded by few genes, were converted significantly less often. The proportions of converted genes related to biological regulation and the regulation of biological processes out of all converted genes were 2.8% and 2.8%, respectively, which was significantly lower than their expected percentages of 6.9% and 6.8% in duplicated genes (*P* value = 0.032, *P* value = 0.036) (Fig. 5a, Additional file 2: Table S13). These results revealed that some genes with specific functions are biased toward conversion, while some functional genes tend to avoid conversion. However, in *S. brachista*, although we also detected that conversion is related to biological function, this correlation did not reach a significant level (Fig. 5b). A possible reason for this phenomenon is that the faster evolutionary rate of *S. brachista* genomes led to duplicates escaping gene conversion.

### Converted genes associated with important traits

To investigate whether the converted genes are involved in specific biological traits, we searched the Non-Redundant Protein Sequence Database (NR) using the converted genes in poplar and willow via the built-in BLASTP program at the NCBI. We summarized the potential biological traits associated with all converted genes, of which 78.6% (423/538) and 81.8% (198/242) of genes in *P. trichocarpa* and *S. brachista* had trait descriptions, respectively (Additional file 2: Table S14-15). We found that transcription factors with conserved structural domains, such as the basic helix-loop-helix protein (bHLH) family, ethylene response factor (ERF) family and CCCH zinc finger family, had the highest frequency. Those genes from transcription factor families often contain conserved functional domains associated with plant phenotypic diversity [60], plant defense responses [61], wood development and drought tolerance regulation [62]; these genes may have been affected by conversion in *P. trichocarpa* and *S. brachista*. In addition, some traits of converted genes in *P. trichocarpa* and *S. brachista* have been reported in poplar and are associated with the functions of glutathione S-transferase and glucuronoxylan methyltransferase (Additional file 2: Table S16). Genes affected by conversion that are also related to transferases or methyltransferases are often associated with stress tolerance, detoxification metabolism and wood synthesis in poplar [63, 64]. These results suggest that the genes with important biological significance and those related to specific traits in *P. trichocarpa* and *S. brachista* may be well conserved and exhibit a high frequency of conversion.

## Discussion

### Long-lasting and extensive conversion between duplicated genes in poplar and willow

The diversification and genetic innovation of Salicaceae species were inextricably related to the contribution of the SCT event [19, 27, 65–67]. Here, by using the representative model species *P. trichocarpa* and *S. brachista* from the family Salicaceae, we offered new insights into the evolution of duplicated genes. First, the 58-million-year-old duplicated genes in poplar and willow experienced numerous gene conversions through homoeologous recombination, which is similar to that in the genomes of rice and sorghum [1, 8, 11], hexaploid wheat [7], Brassica [10], *Gossypium* [5], and *Arachis* [9, 68]. Second, the rate of conversion in poplar was approximately twice that in willow, which may be because the poplar genome was more conserved and preserved more SCT-produced duplicated genes [19, 27]. Third, the duplicated genes in poplar and willow were mainly affected by partial-gene conversion, but only a very limited number of genes were affected by whole-gene conversion. This phenomenon could be explained by the extensive chromosomal rearrangements, resulting in only occasional small-scale conversion events [1, 26, 27, 66, 69]. In contrast, rice and sorghum have fewer genomic structure changes than the grass ancestor karyotype and have shown a large proportion of whole-gene conversion in previous studies [1, 8, 70, 71]. Additionally, in this study, we detected some duplicated gene pairs affected by conversion at a very recent time, such as the paralogs *Ptr10g2249*-*Ptr8g0063* and *Ptr3g1166*-*Ptr1g0855,* which have *Ks* values close to zero. Therefore, we emphasized that conversion in the poplar and willow genomes is a long-lasting and continuous DNA recombination process, which is similar to what occurs in the genomes of rice, sorghum, *Arachis*, and *Oryza* [1, 8, 9, 11, 47, 68].

### Well-preserved genomic colinear regions are easily converted

The duplicated genes distributed near the termini of chromosomes were preferentially affected by gene conversion, which has been proposed in rice and sorghum genomes [1, 8, 47]. In this study, we found that the converted duplicates were unevenly distributed on each chromosome, there was no evidence of preferential conversion near the ends of the chromosomes in poplar and willow. This result seems unreasonable because the genes near the chromosomal termini are usually well conserved and can provide more possibilities for conversion based on sequence similarity [1, 8, 11, 72]. We speculate that this result was also attributed to the complex genomic rearrangements of poplar and willow, where the structure of ancestral chromosomes was altered by the insertion of duplicated segments into the telomeric and centromeric regions of other chromosomes after the SCT [66]. In other words, the terminal regions of ancestral chromosomes in poplar and willow may no longer be located close to the telomeres. This result coincides with our subsequent finding that the length of the blocks (colinear gene pairs) was positively correlated with the conversion rate, i.e., the well-preserved homoeologous regions showed a higher conversion rate.

### Gene conversion accelerated the evolutionary rate and species divergence

Gene conversion is one of the two mechanisms of homologous recombination [2]. And homologous recombination is the main driver of genetic innovation within an organism [12, 33]. Therefore, gene conversion may play an important role in species divergence. A hallmark of its role is that in two closely related species, paralogous gene sequences become more closely related to each other than they are to their orthologous [73]. This conclusion is further supported by this study in poplar and willow, where we found that the *Ks* between converted paralogs was smaller than those between nonconverted paralogs, and the *Ks* for converted orthologs was significantly larger than the nonconverted orthologs. These converted genes show relatively small sequence divergence and appear younger, but actually evolve faster. This can be explained by a classical evolutionary theory, which anticipates that gene redundancy may lead to a relatively rapid accumulation of variation and thus facilitate gene evolution [74]. We therefore suggest that gene conversion is essentially accelerated the rate of evolution and increased species divergence, which has been demonstrated by comparison of the duplicated genes in rice and sorghum [1, 2, 47]. Furthermore, by comparing *Pn*/*Ps*, we found that the converted genes had larger *Pn*/*Ps* than those that unconverted, suggesting that purifying selection was reduced by conversion in both poplar and willow. Although it is possible that conversion did not actually affect selection pressure, and gene conversion may have occurred only within these highly conserved genes. The conserved nature of these genes leads to the occurrence of gene conversions between homologues, rather than conversion promoting gene conservation.

### Multigene families and conserved genes may be preferentially converted

Gene conversion seems to favor some alleles over others, a process known as biased gene conversion [2]. In this study, we confirmed that members of large gene families are more biased towards conversion. The evolution of functional genes that are members of these multigene families is often accompanied by strong positive selection [75–78]. These results are consistent with previous studies, which suggested that most multigene families were thought to have coevolved with related homologs through gene conversion and that members of these multigene families are more likely to undergo more gene conversions [79, 80]. And in these families, most genes are usually closely related and extremely similar. They usually encode conserved structural domains and are mainly involved in transcriptional regulation and resistance to biotic or abiotic stresses. In addition, as mentioned above, converted genes are under less selective pressure. Although some researchers have suggested that gene conversion may be conserved to repair deleterious mutations, it is more likely that gene conversion has facilitated the spread of multigene family members or favorable mutations.

## Conclusion

Duplicated genes produced by polyploidization were converted in poplar and willow genomes. By performing comparative genomics and phylogenetics, we identified the scale and patterns of conversion between duplicates produced by the SCT during diversification in poplar and willow. Gene conversion maintained the similarity between duplicated sequences, providing the opportunity for further gene conversion and accelerating the evolutionary rate of poplar and willow. Chromosomal rearrangements following polyploidization were associated with gene conversion, and well-preserved regions on homoeologous chromosomes may facilitate duplicate conversion. Converted duplicates had more similar expression patterns. In terms of biological function, genes associated with multigene families may be preferentially converted. Genes containing conserved structural domains, which are associated with specific and important functional traits, may be converted more frequently. Our findings contribute to the understanding of the evolution of converted genes in poplar and willow.

## Methods

### Inferring gene collinearity

To identify the duplicated genes produced by the SCT and the orthologous genes related to the speciation of the considered genomes, we first searched for potential homologous gene pairs by using BLASTP [57], with the strict parameters of e-value < 1E-5 and Score > 100. Then, gene homology information was used as input into ColinearScan [58] to locate the colinear gene pairs and test the significance of the collinearity of chromosomal regions (blocks). Here, the key parameter, the maximum gap, was set to 50 intervening genes; large gene families with 50 or more members were removed from the blocks. Finally, we performed genomic homologous structure analyses through homologous dot plots to help determine the paralogous and orthologous genes. This genome collinearity analysis approach has been adopted in many previous angiosperm genomic comparisons [8, 41, 81].

### Calculation of Ks and Ka

The synonymous nucleotide substitution rate (*Ks*) and nonsynonymous nucleotide substitution rate (*Ka*) between homologous gene pairs were estimated by using the Nei-Gojobori [82] approach with the program codeml in PAML [83]. ClustalW was employed to align multiple gene CDSs and set the default parameters [84]. Because nucleotide substitutions may frequently occur at the same site in a sequence, we used the Jukes-Cantor (JC) model to correct the *Ka* and *Ks* values, denoted as *Pn* and *Ps* [1, 85].

### Kernel function analysis of Ks

*Ks* values of homologous genes from different genomes can reflect the time of divergence and speciation. We used kernel function to analyze the *Ks* distribution of colinear homologs within and between genomes. The *Ks* distribution is thought of as a mix of normal distributions. The width of the kernel smoothing density function of *Ks* is set to 0.05 using MATLAB [86] to estimate the density of each *Ks* list and obtain the density distribution curves. The curve was Gaussian fitted by the fitting toolbox cftool. The parameter *R-squared* is used to evaluate the fitting goodness and is generally set to at least 95%; the smallest number of normal distributions was used to represent the complex *Ks* distribution; and the corresponding evolutionary event is represented by principle one.

### Topology tree construction

To clarify polyploidization events of selected genomes, we first used MEGA-X [87] to construct topology trees with homologous genes from multiple genome alignment lists. The maximum likelihood algorithm was employed to construct the gene trees, and the bootstrap value was set to 1,000 to ensure the stability of evaluation.

### Construction of homologous gene quartets

We used multiple sequence alignments to construct homologous gene quartets based on the homologous relationships. By checking colinear genes in the multiple sequence alignments, the paralogous gene pairs in each of the involved genomes and the orthologous gene pairs between genomes were obtained, and homologous gene quartets were retrieved. Assuming that both *P. trichocarpa* and *S. brachista*, P and S, retain a pair of duplicated chromosomal segments generated in their common ancestor through polyploidization, then the paralogous genes P1 and P2 and their respective orthologous genes S1 and S2 comprise a homologous gene quartet.

### Inference of gene conversion

With each gene quartet, multiple sequence alignment was performed using ClustalW [59]. We removed highly scattered tetrads to eliminate potential problems caused by inferring gene conversion from unreliable sequences. We removed quartets showing gaps in pairwise comparisons of more than 50% of the length and those with less than 40% amino acid homology between homologous sequences.

Whole-gene conversion (WCV) inference: Because homologs arose prior to subspecies divergence, we expected that homologs between the two subspecies should be more similar to each other than to those within each subspecies. If the analogs of different subspecies are more similar to each other than to their respective homologs, we infer that gene conversion occurred after species divergence. To measure the similarity of homologous genes in each quadruplet, we characterized the *Ks* values and amino acid site identity ratios between paralogs and orthologs. *Ks* values between paralogous and orthologous gene pairs were used to infer possible whole-gene conversion.

Partial-gene conversion (PCV) inference: Possible gene conversion due to partial-gene conversion after species divergence was identified using quartets. A combination of dynamic programming and phylogenetic analysis was used to document the differences between two aligned bases from intragenomic and intergenomic homologs [1]. Thus, inferring gene conversion involves 5 steps. 1) Defining data to reflect the distance between homologs. 2) Averaging the distance arrays of direct homologous gene pairs and comparing the average distance between paralogous homologs and direct homologs, since paralogous homologs should be more distant if PCV is not involved. 3) Inferring the extended range of paralogous homologs using dynamic programming to reveal high-scoring fragment sequences, followed by identifying partially affected regions ≥ 10 nucleotides in length. 4) Identifying the high scoring fragments with shorter lengths and smaller scores based on bootstrap tests. 5) After masking some larger fragments, performing recursive procedures to reveal shorter, high scoring fragments, which helped to reveal genes affected by multiple gene conversion events. The scripts of gene conversions inference have deposited in Github (https://github.com/wangjiaqi206/gene-conversion), more detailed information also can be found in the previous article [1, 8, 48].

### Duplicated gene density and conversion rate

To reveal the relationship between duplicated genes and the gene conversion rate, we counted and analyzed the density of duplicated genes and the conversion rate. The duplicated genes on each chromosome were divided into small fragments of 1 Mb from the anterior and terminal ends of the chromosome to the center. Then, the number of duplicates and converted genes in each fragment were counted. Gene density was calculated by dividing the number of duplicated genes by the number of all genes in each fragment. The conversion rate was calculated by dividing the number of converted genes by the number of duplicated genes in each fragment. Duplicated genes here referred to those generated from the SCT. Finally, we used smoothed curves, which were marked with Pearson correlation coefficients, to characterize the correlation between gene density and the replacement rate.

### Conversion and gene ontology analysis

To obtain an overview of the function of duplicated genes, InterProScan5 [88] was used to determine the GO classification of each gene. All records are derived from literature-based annotations and domain-based electronic annotations. GO annotation results for the transformed and untransformed gene sets were compared and plotted using the online visualization tool WEGO [89] to visualize the distribution and trends of functional genes. The significance of the difference in the number of functional duplicates between converted and nonconverted genes was tested by Pearson’s chi-square test.

### Differential expression of converted genes

We processed the raw RNA-seq reads using Trimmomatic software [90] and removed the adaptor sequences and low-quality reads with the default parameters. These transcriptomes of *P. trichocarpa* secondary xylem (SRR13481183) and *S. brachista* leaves (SRR7341541) were downloaded from GenBank. We then mapped clean reads to the genomes of *P. trichocarpa* and *S. brachista* by using Hisat2 software [91] with default parameters, and quantified them by using StringTie software [92] with the '-e -A' parameter. In addition, to analyze the expression patterns of converted and nonconverted genes, we removed the nonexpressed genes and selected TPM expression abundance as the reference. The homogenization process of TPM results in the same overall expression across different samples, which was ideal for this study.

### Presumed biological functions for converted genes

To identify the possible biological functions of the converted genes, we first performed an online BLASTP homology comparison of the obtained genes in GenBank. We then filtered the results to prioritize the homologous genes with ≥ 99% sequence similarity to the subjects and recorded the function or trait of these selected genes. In addition, some homologous genes have been previously reported in the literature, and we counted them accordingly.

## Supplementary Information

**Additional file 1: Fig. S1. **Genomic comparisons of the studied genomes. a Intergenomic comparison of the *V. vinifera* and *P. trichocarpa* genome. b Intergenomic comparison of the *V. vinifera* and *S. brachista* genome; c Intragenomic comparison within the *P. trichocarpa* genome; d Intergenomic comparison of the *S. brachista* and *P. trichocarpa* genome. Best-hit genes are represented by red dots and other genes by gray dots. The grape 19 chromosomes colored by 7 eudicot ancestral chromosomes. Genomic syntenic blocks (≥8 gene pairs) inferred from ColinearScan are shown in dotplot according to the genomic locations of *V. vinifera*, *P. trichocarpa*, and *S. brachista*. In a and b, the solid boxes indicate the orthologous regions produced by core-eudicot-common hexaploidization (ECH). In c, the solid boxes indicate the paralogous regions produced by SCT (Salicaceae common tetraploidization event). In d, the solid boxes indicate the orthologous regions between *S. brachista* and *P. trichocarpa*. The best paralogy or orthology ratios between studied genomes were 1:2, 1:2, 1:1 and 2:2 in a, b, c and d, respectively. **Fig. S2. **Intragenomic comparison of the *S. brachista* genome. The homologous gene dotplot within *S. brachista* showed the best paralogy ratio of 1:1. Detailed notation and explanation can be found in the legend of Fig. S1. **Figure S3. **Intragenomic comparison analyses of *P.trichocarpa *with* P. trichocarpa*. The best and other matched homologous gene pairs are shown by red and gray dots, respectively. Mean *Ks* of each inferred colinear blocks are exhibited near their corresponding regions. **Fig. S4. **Intragenomic comparison analyses of *S. brachista *with* S. brachista*. The best and other matched homologous gene pairs are shown by red and gray dots, respectively. Mean *Ks* of each inferred colinear blocks are exhibited near their corresponding regions. **Fig. S5. **Intergenomic comparison analyses of *P. trichocarpa *with* S. brachista*. The best and other matched homologous gene pairs are shown by red and gray dots, respectively. Mean *Ks* of each inferred colinear blocks are exhibited near their corresponding regions. **Fig. S6. **The correction of conversion and density of duplicates in *P. trichocarpa*. The Y-axis indicatesthe density of duplicated genes (blue lines) and conversion rate (green lines) for selected chromosomes in *P. trichocarpa*. The X-axis indicates the distance of the duplicated or converted genes from the chromosome termini. **Fig. S7.** The correction of conversion and density of duplicates in *S. brachista*. The Y-axis indicates the density of duplicated genes (blue lines) and conversion rate (green lines) for selected chromosomes in *S. brachista*. The X-axis indicates the distance of the duplicated or converted genes from the chromosome termini.

**Additional file 2: Table S1. **Number of homologous blocks and gene pairs within a genome or between genomes. **Table S2.** Kernel function analysis of *Ks* distribution related to duplication events within each genome and between selected genomes. **Table S3.** Identified quartets and gene conversion in *P. trichocarpa* and *S. brachista*. **Table S4.** Gene conversion and quartets in *P. trichocarpa* and *S. brachista*. **Table S5.** Gene conversion and quartets in  *S. brachista* and *P. trichocarpa*. **Table S6.** Paralogous gene conversion physical location in *P. trichocarpa* and *S. brachista*. **Table S7.** The correction of conversion and density of duplicates in *P. trichocarpa*. **Table S8.** The correction of conversion and density of duplicates in *S. brachista*. **Table S9.** Expression level of converted and non-converted gene pairs in *P. trichocarpa* and *S. brachista*. **Table S10.** Comparison of expression differences between converted and non-converted gene pairs. **Table S11.** Comparison of mean TPM difference between converted and non-converted gene pairs. **Table S12.** Gene ontology analysis of duplicated genes. **Table S13.** GO analysis of converted genes and duplicates in *P. trichocarpa* and *S. brachista*. **Table S14.** The possible specific biological traits of converted genes in *P. trichocarpa*. **Table S15.** The possible specific biological traits of converted genes in *S. brachista*. **Table S16.** The possible biological traits of reported converted genes  in *P. trichocarpa* and *S. brachista*. **Table S17.** Information of original data material.

## Data Availability

The datasets supporting the conclusions of this article are included within the article and its additional files. Genomes used in the article were downloaded from their various databases, *Vitis vinifera* (https://phytozome-next.jgi.doe.gov/info/Vvinifera_v2_1) and *Populus trichocarpa* (https://phytozome-next.jgi.doe.gov/info/Ptrichocarpa_v4_1) were downloaded from the Phytozome (https://phytozome-next.jgi.doe.gov/), *Salix brachista* (accession number ASM907833v1, https://www.ncbi.nlm.nih.gov/genome/?term=ASM907833v1) were downloaded from the GenBank (https://www.ncbi.nlm.nih.gov/) (Additional file 2: Table S17). Transcriptome data of *P. trichocarpa* secondary xylem (accession number SRR13481183, https://www.ncbi.nlm.nih.gov/sra/SRR13481183/) and *S. brachista* leaves (accession number SRR7341541, https://www.ncbi.nlm.nih.gov/sra/?term=SRR7341541) were downloaded from GenBank (https://www.ncbi.nlm.nih.gov/).

## Abbreviations

MYA: Million years ago
ECH: Core-eudicot-common hexaploidization event
SCT: Salicaceae common tetraploidization
WCV: Whole-gene conversion
PCV: Partial-gene conversion
