# Supplementary material for: Conversion between duplicated genes generated by polyploidization contributes to the divergence of poplar and willow
Source: BMC Plant Biol. 2022 Jun 17;22:298. doi: 10.1186/s12870-022-03684-9 (PMC9205023; doi:10.1186/s12870-022-03684-9)
Supplement: Supplementary file 1 — Additional file 1: Fig. S1. Genomic comparisons of the studied genomes. a Intergenomic comparison of the V. vinifera and P. trichocarpa genome. b Intergenomic comparison of the V. vinifera and S. brachista genome; c Intragenomic comparison within the P. trichocarpa genome; d Intergenomic comparison of the S. brachista and P. trichocarpa genome. Best-hit genes are represented by red dots and other genes by gray dots. The grape 19 chromosomes colored by 7 eudicot ancestral chromosomes. Genomic syntenic blocks (≥8 gene pairs) inferred from ColinearScan are shown in dotplot according to the genomic locations of V. vinifera, P. trichocarpa, and S. brachista. In a and b, the solid boxes indicate the orthologous regions produced by core-eudicot-common hexaploidization (ECH). In c, the solid boxes indicate the paralogous regions produced by SCT (Salicaceae common tetraploidization event). In d, the solid boxes indicate the orthologous regions between S. brachista and P. trichocarpa. The best paralogy or orthology ratios between studied genomes were 1:2, 1:2, 1:1 and 2:2 in a, b, c and d, respectively. Fig. S2. Intragenomic comparison of the S. brachista genome. The homologous gene dotplot within S. brachista showed the best paralogy ratio of 1:1. Detailed notation and explanation can be found in the legend of Fig. S1. Figure S3. Intragenomic comparison analyses of P.trichocarpa with P. trichocarpa. The best and other matched homologous gene pairs are shown by red and gray dots, respectively. Mean Ks of each inferred colinear blocks are exhibited near their corresponding regions. Fig. S4. Intragenomic comparison analyses of S. brachista with S. brachista. The best and other matched homologous gene pairs are shown by red and gray dots, respectively. Mean Ks of each inferred colinear blocks are exhibited near their corresponding regions. Fig. S5. Intergenomic comparison analyses of P. trichocarpa with S. brachista. The best and other matched homologous gene pairs are shown by [file 12870_2022_3684_MOESM1_ESM.docx]

**Additional figures**


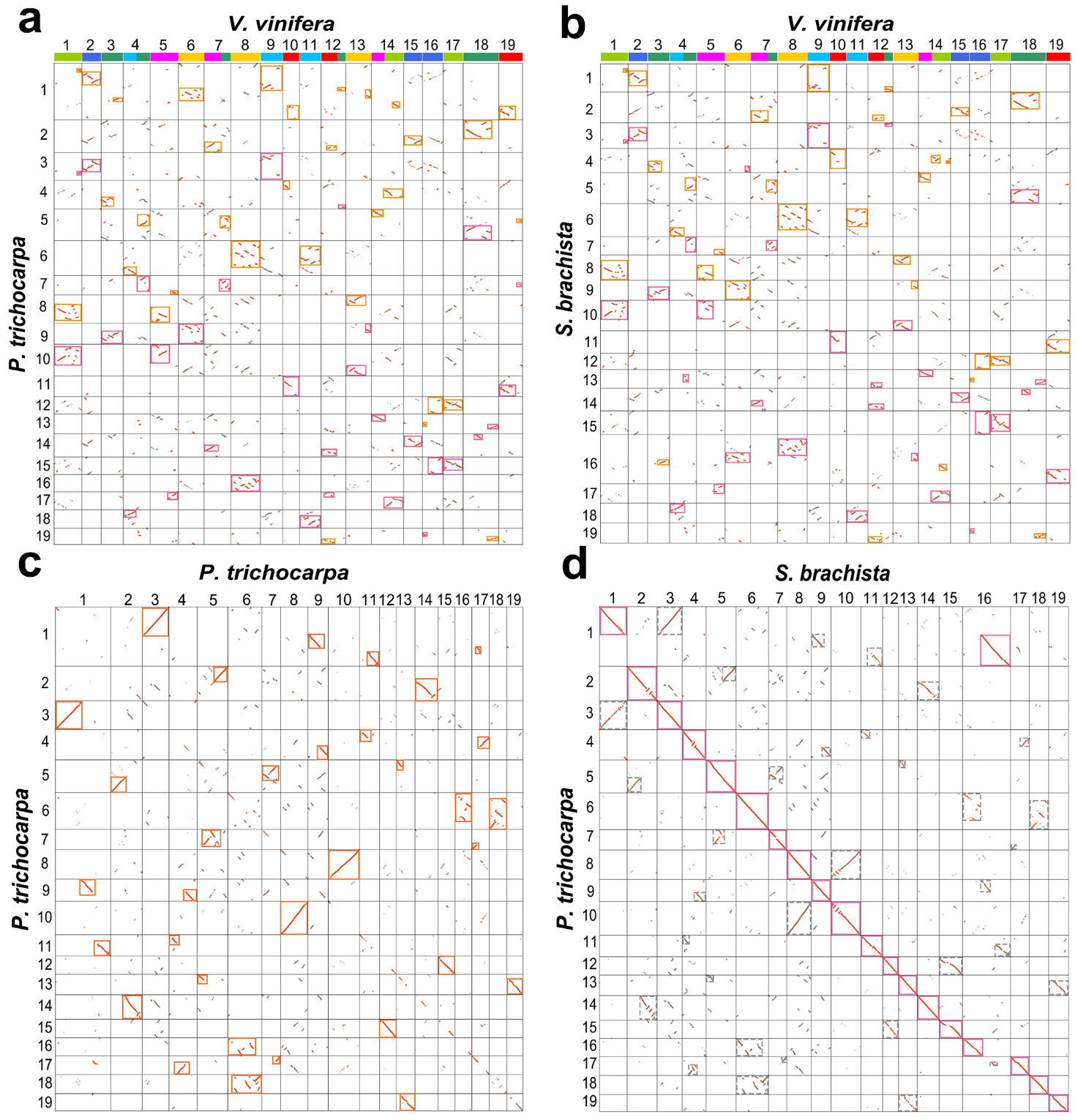


**Fig.** **S1. Genomic comparisons of the studied genomes.**

**a** Intergenomic comparison of the *V. vinifera* and *P. trichocarpa* genome. **b** Intergenomic comparison of the *V. vinifera* and *S. brachista* genome; **c** Intragenomic comparison within the *P. trichocarpa* genome; **d** Intergenomic comparison of the *S. brachista* and *P. trichocarpa* genome. Best-hit genes are represented by red dots and other genes by gray dots. The grape 19 chromosomes colored by 7 eudicot ancestral chromosomes. Genomic syntenic blocks (≥8 gene pairs) inferred from ColinearScan are shown in dotplot according to the genomic locations of *V. vinifera, P. trichocarpa,* and *S. brachista*. In **a** and **b,** the solid boxes indicate the orthologous regions produced by core-eudicot-common hexaploidization (ECH). In **c,** the solid boxes indicate the paralogous regions produced by SCT (Salicaceae common tetraploidization event). In **d**, the solid boxes indicate the orthologous regions between *S. brachista* and *P. trichocarpa*. The best paralogy or orthology ratios between studied genomes were 1:2, 1:2, 1:1 and 2:2 in **a**, **b**, **c** and **d**, respectively.


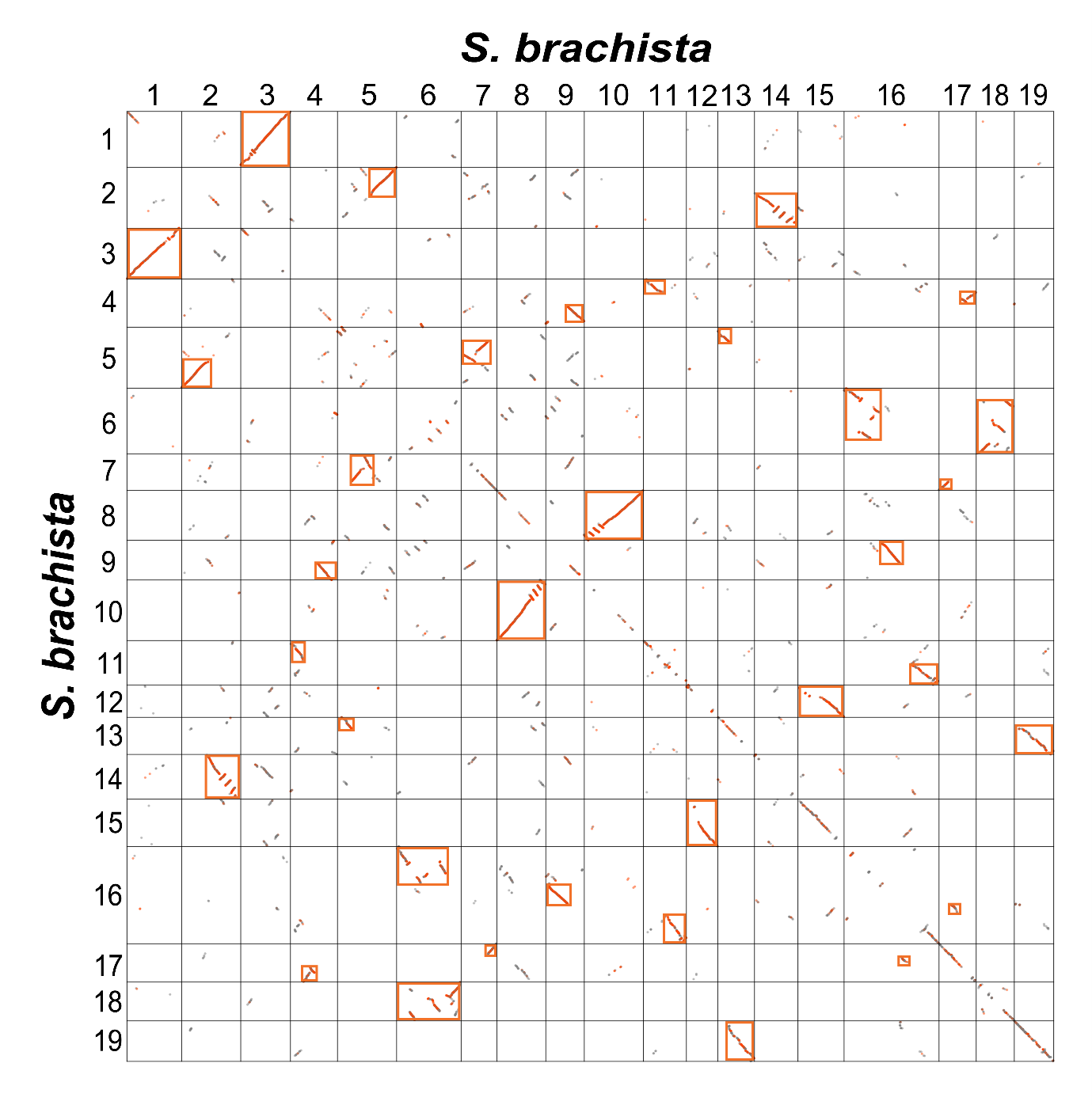


**Fig. S2. Intragenomic comparison of the *S. brachista* genome.**

The homologous gene dotplot within *S. brachista* showed the best paralogy ratio of 1:1. Detailed notation and explanation can be found in the legend of Fig. S1.


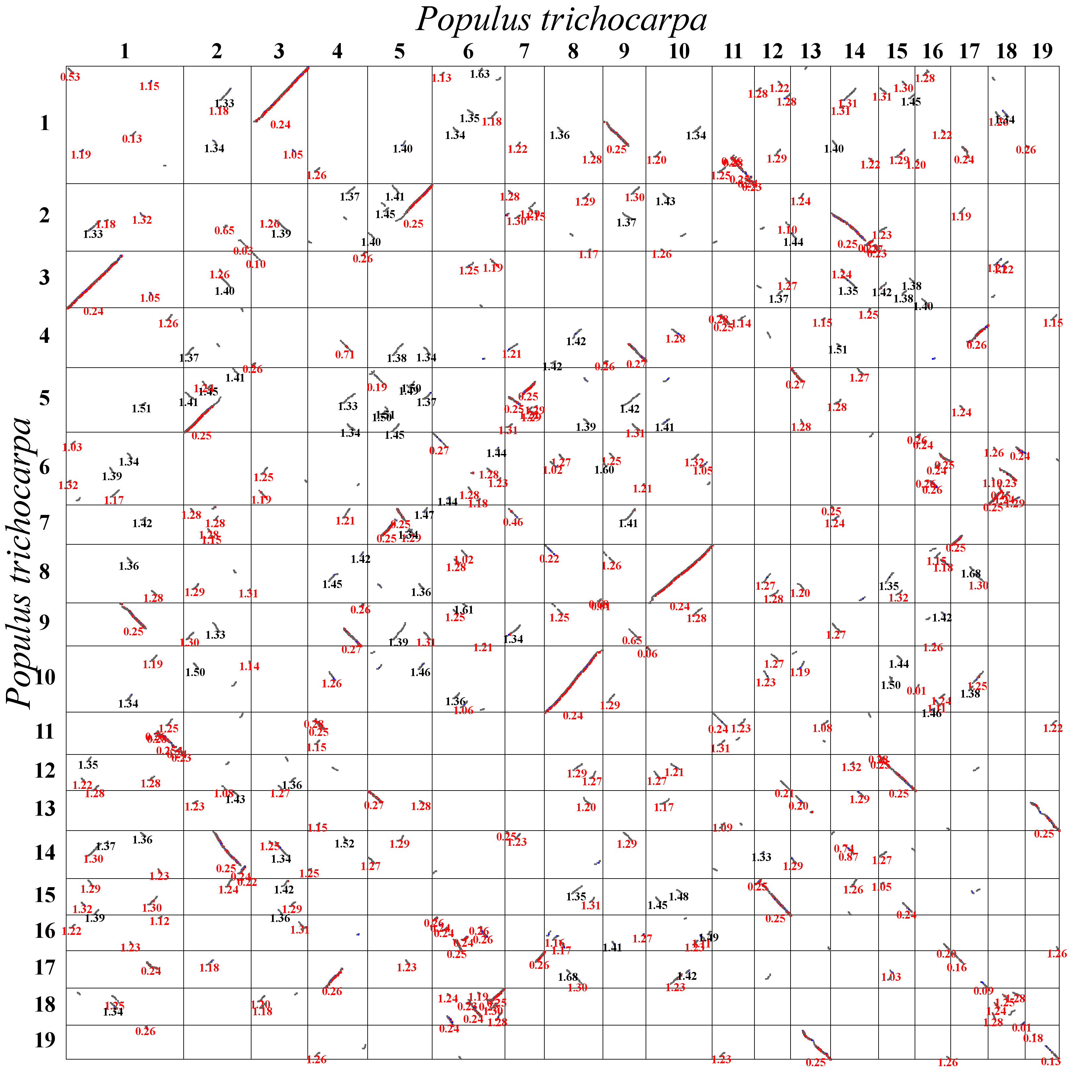


**Figure S3. Intragenomic comparison analyses of *P. trichocarpa* with *P. trichocarpa*.**

The best and other matched homologous gene pairs are shown by red and gray dots, respectively. Mean *Ks* of each inferred colinear blocks are exhibited near their corresponding regions.


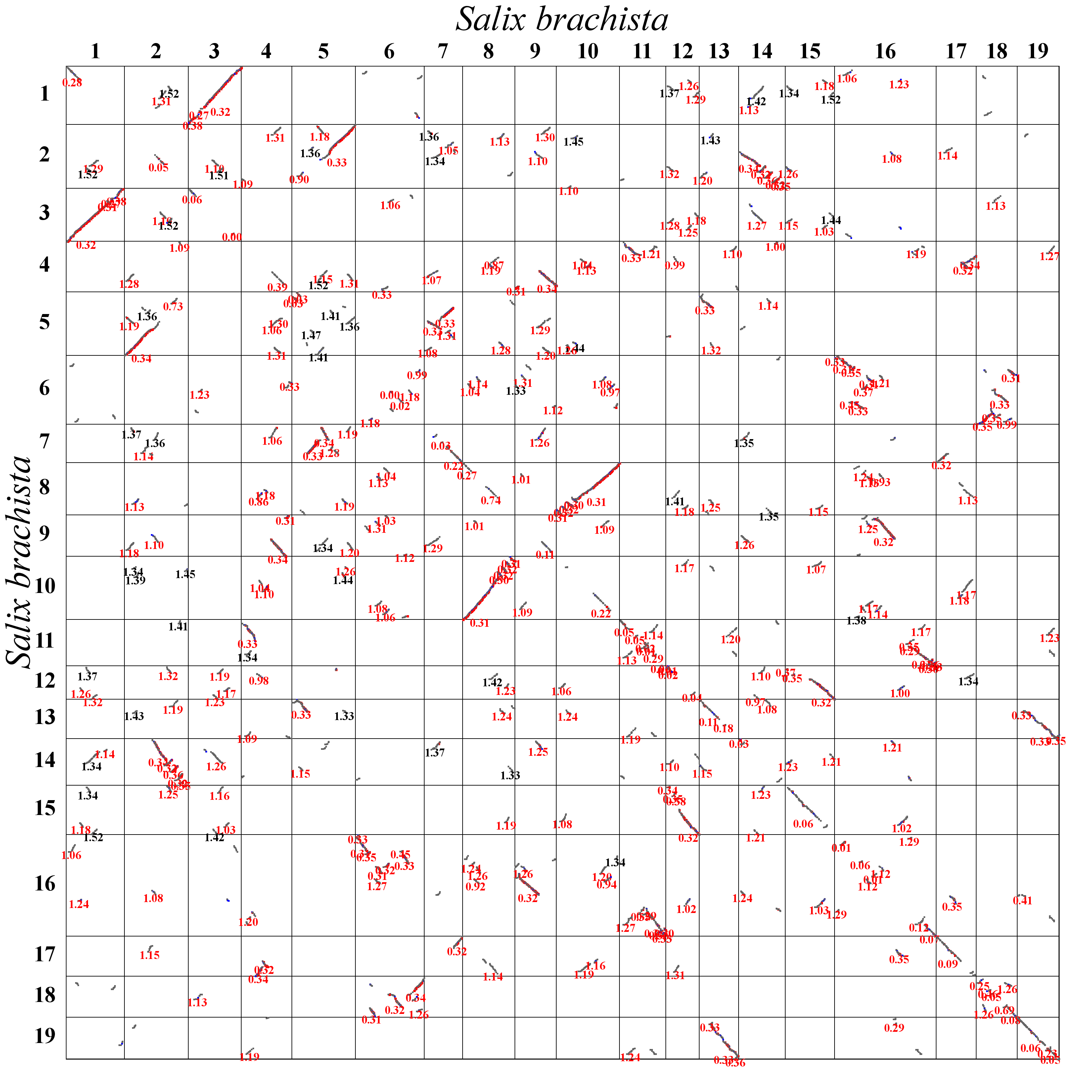


**Fig. S4. Intragenomic comparison analyses of *S. brachista* with *S. brachista*.**

The best and other matched homologous gene pairs are shown by red and gray dots, respectively. Mean *Ks* of each inferred colinear blocks are exhibited near their corresponding regions.


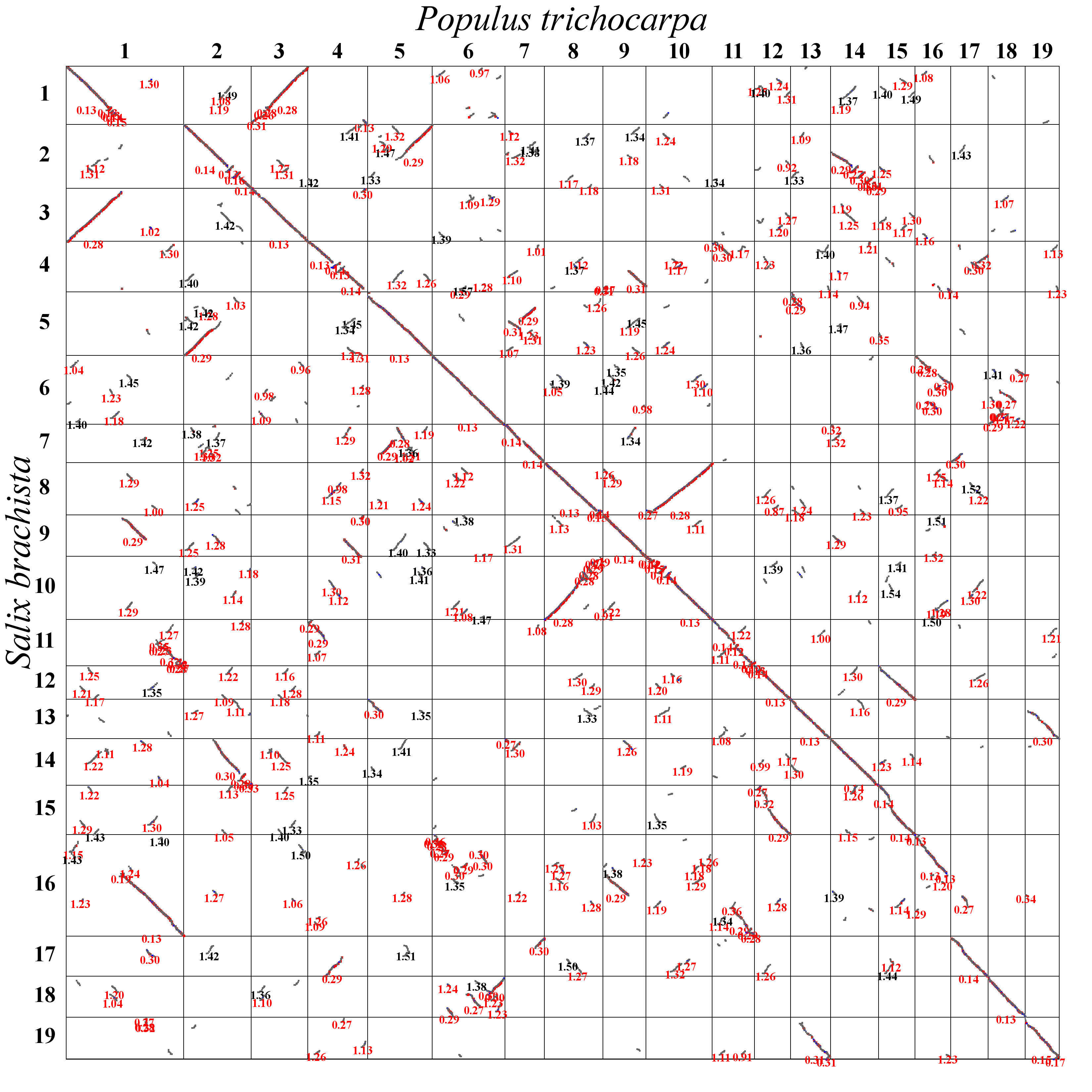


**Fig. S5. Intergenomic comparison analyses of *P. trichocarpa* with *S. brachista*.**

The best and other matched homologous gene pairs are shown by red and gray dots, respectively. Mean *Ks* of each inferred colinear blocks are exhibited near their corresponding regions.


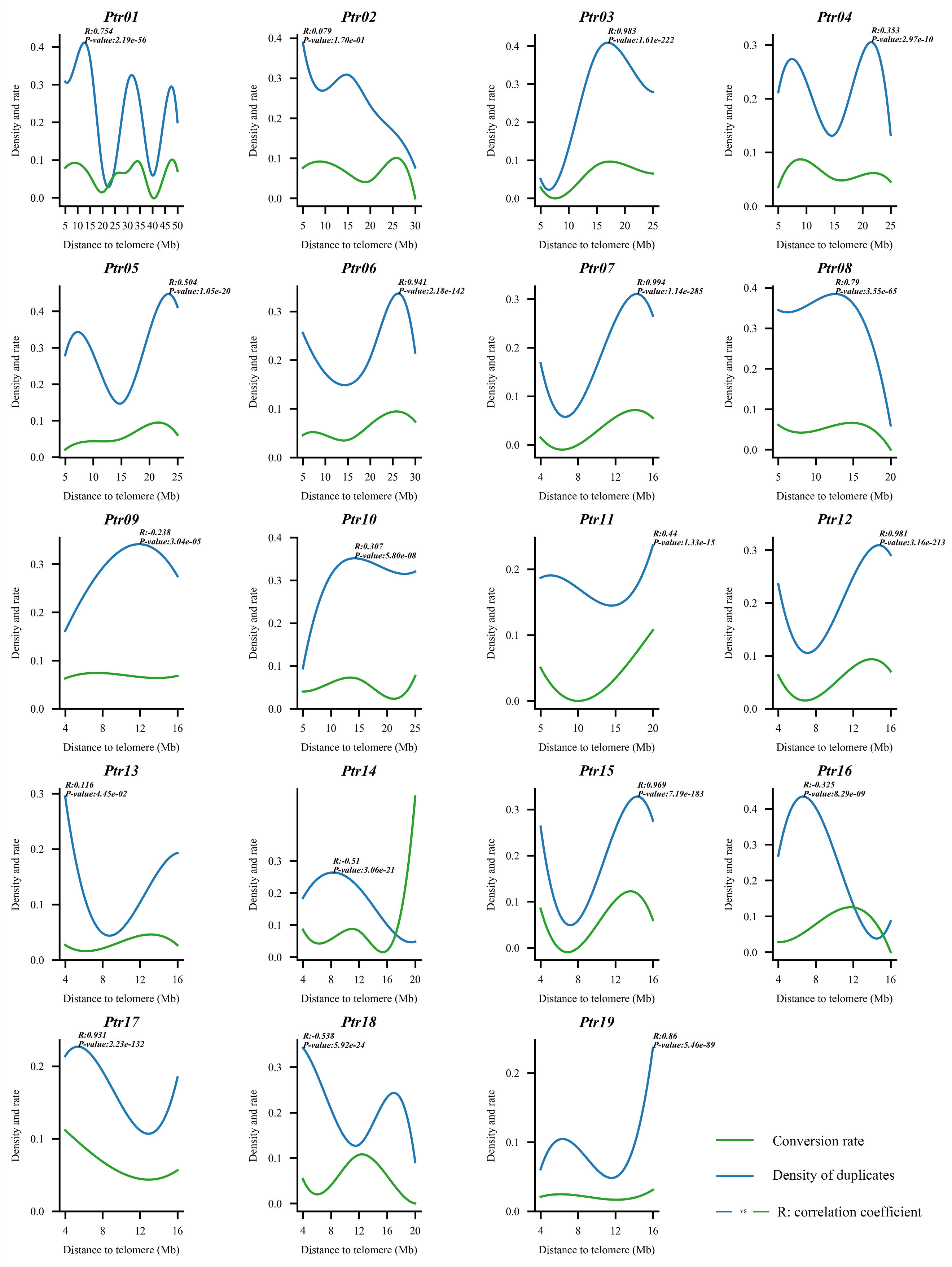


**Fig. S6. The correction of conversion and density of duplicates in *P. trichocarpa*.**

The Y-axis indicates the density of duplicated genes (blue lines) and conversion rate (green lines) for selected chromosomes in *P. trichocarpa*. The X-axis indicates the distance of the duplicated or converted genes from the chromosome termini.


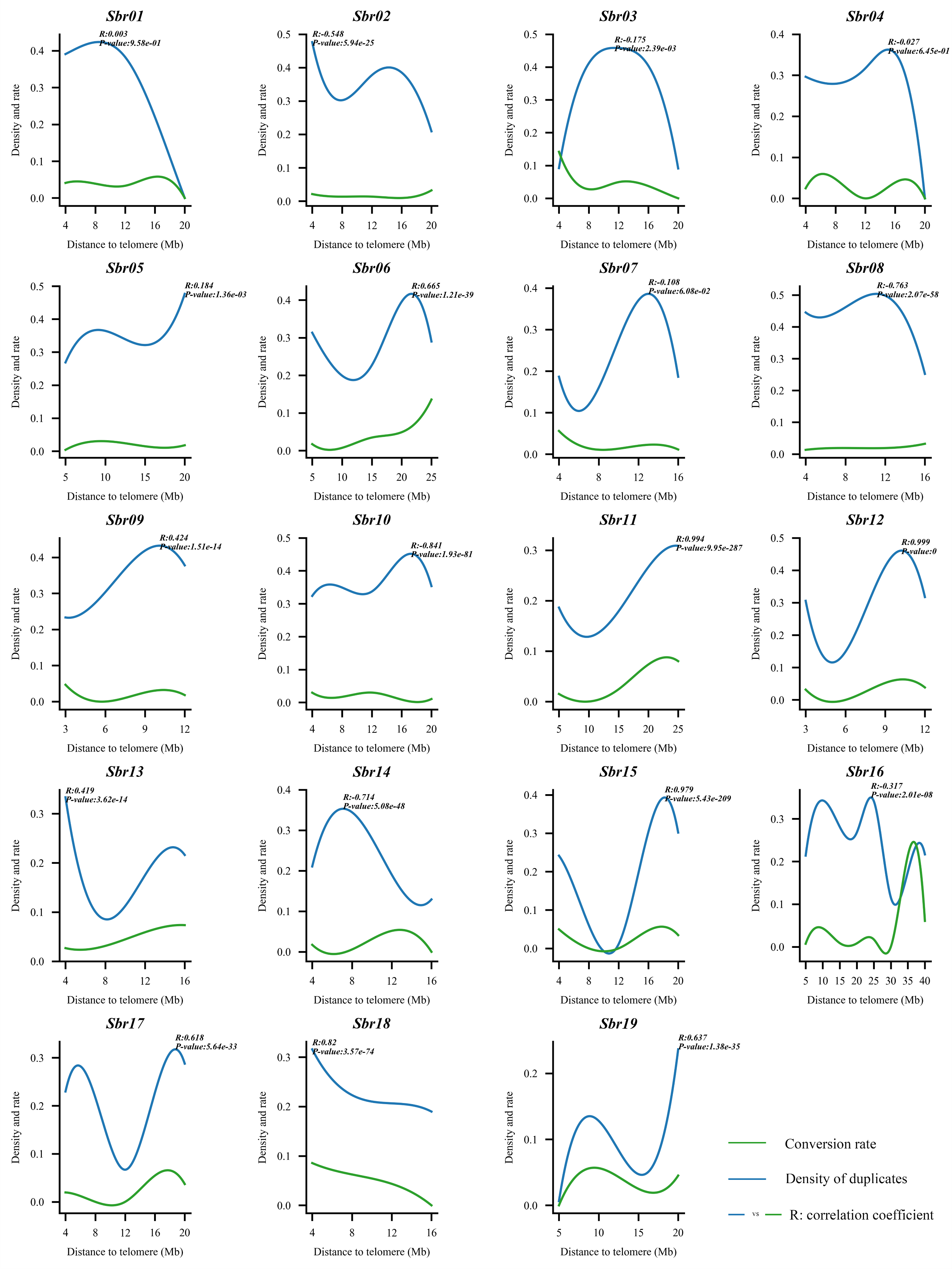


**Fig. S6. The correction of conversion and density of duplicates in *S. brachista*.**

The Y-axis indicates the density of duplicated genes (blue lines) and conversion rate (green lines) for selected chromosomes in *S. brachista*. The X-axis indicates the distance of the duplicated or converted genes from the chromosome termini.
